# Supplementary material for: Changes in BNP levels from discharge to 6-month visit predict subsequent outcomes in patients with acute heart failure
Source: PLoS One. 2022 Jan 28;17(1):e0263165. doi: 10.1371/journal.pone.0263165 (PMC8797237; doi:10.1371/journal.pone.0263165)
Supplement: S1 Table — Categorical variables are presented as number (%), and continuous variables are presented as mean ± SD. BNP, brain natriuretic peptide; BMI, body mass index; AF, atrial fibrillation; AFL, atrial flutter; eGFR, estimated glomerular filtration rate; ACE-I, angiotensin converting-enzyme inhibitor; ARB, angiotensin-receptor blocker; MRA, mineralocorticoid receptor antagonist; SD, standard deviation. Diuretics included loop diuretic, thiazide or tolvaptan. (PDF) [file pone.0263165.s001.pdf]

**S1 Table. Patient characteristics compared between patients with available data on change in BNP and those without data**

| Variables | BNP change (N=446) | BNP change loss (N=678) | P value |
|---|---|---|---|
| Age (years) | 74.8 ± 12.4 | 76.4 ± 12.3 | 0.04 |
| Age ≥80 years | 195 (44 %) | 309 (46 %) | 0.54 |
| Women | 209 (47 %) | 265 (39 %) | 0.001 |
| BMI (kg/m$^2$) | 22.4 ± 5.1 | 21.4 ± 3.9 | 0.59 |
| BMI ≤22 kg/m$^2$ | 241 (56 %) | 395 (61 %) | 0.29 |
| AF or AFL | 269 (60 %) | 358 (53 %) | 0.01 |
| Hypertension | 327 (73 %) | 497 (73 %) | 1.00 |
| Diabetes | 166 (37 %) | 265 (39 %) | 0.53 |
| Previous myocardial infarction | 101 (23 %) | 151 (22 %) | 0.88 |
| eGFR (mL/min/1.73m$^2$) | 45.3 ± 20.2 | 44.6 ± 20.2 | 0.64 |
| eGFR <30 mL/min/1.73m$^2$ | 108 (24 %) | 97 (26 %) | 0.50 |
| Albumin (g/dL) | 3.9 ± 0.54 | 3.9 ± 0.49 | 0.07 |
| Albumin <3 g/dL | 17 (4.0 %) | 11 (3.4 %) | 0.65 |
| Hemoglobin (g/dL) | 12.0 ± 2.1 | 12.0 ± 2.3 | 0.84 |
| Anemia | 258 (59 %) | 223 (61 %) | 0.57 |
| ACE-I or ARB | 226 (63 %) | 194 (55 %) | 0.02 |
| β-blocker | 279 (78 %) | 253 (72 %) | 0.06 |
| MRA | 162 (46 %) | 157 (45 %) | 0.86 |
| Diuretics | 303 (85 %) | 289 (82 %) | 0.32 |

Categorical variables are presented as number (%), and continuous variables are presented as mean ± SD.

BNP, brain natriuretic peptide; BMI, body mass index; AF, atrial fibrillation; AFL, atrial flutter; eGFR, estimated glomerular filtration rate; ACE-I, angiotensin converting-enzyme inhibitor; ARB, angiotensin-receptor blocker; MRA, mineralocorticoid receptor antagonist; SD, standard deviation.

Diuretics included loop diuretic, thiazide or tolvaptan.
