# Supplementary material for: Changes in BNP levels from discharge to 6-month visit predict subsequent outcomes in patients with acute heart failure
Source: PLoS One. 2022 Jan 28;17(1):e0263165. doi: 10.1371/journal.pone.0263165 (PMC8797237; doi:10.1371/journal.pone.0263165)
Supplement: S2 Table — The Cox proportional hazards regression model was constructed adjusting for 10 clinically relevant risk-adjusting variables: age, LVEF, BNP at discharge, eGFR and albumin as a continuous variable and sex, diuretics, ACE-I or ARB, β-blocker and MRA. Diuretics included loop diuretic, thiazide and tolvaptan. LVEF, left ventricular ejection fraction; BNP, brain natriuretic peptide; eGFR, estimated glomerular filtration rate; ACE-I, angiotensin-converting enzyme inhibitor; ARB, angiotensin-receptor blocker; MRA, mineralocorticoid receptor antagonist; HR, hazard ratio; CI, confidence interval. (PDF) [file pone.0263165.s002.pdf]

S2 Table. Sensitivity analyses

Clinical outcome measures	Categorized group	N of patients with event/N of patients at risk (Cumulative 180-day incidence)	Adjusted HR (95% CI)	P value
**Primary outcome measure (a composite of all-cause death or hospitalization for heart failure)**				
	BNP worsening	39/101 (26.8 %)	3.47 (1.46-8.21)	0.005
	No-marked BNP change	21/119 (14.4 %)	1.92 (0.80-4.59)	0.14
	Marked BNP improvement	10/129 (6.9%)	1 (Reference)	
**All-cause death**				
	BNP worsening	14/125 (9.6 %)	1.94 (0.52-7.27)	0.32
	No-marked BNP change	7/133 (4.8 %)	2.14 (0.58-7.93)	0.26
	Marked BNP improvement	6/133 (4.1%)	1 (Reference)	
**Hospitalization for heart failure**				
	BNP worsening	30/101 (21.2 %)	5.19 (1.59-17.0)	0.007

	No-marked BNP change	14/119 (9.9 %)	1.85 (0.54-6.29)	0.33
	Marked BNP improvement	4/129 (2.8%)	1 (Reference)	

The Cox proportional hazards regression model was constructed adjusting for 10 clinically relevant risk-adjusting variables: age, LVEF, BNP at discharge, eGFR and albumin as a continuous variable and sex, diuretics, ACE-I or ARB, β-blocker and MRA.

Diuretics included loop diuretic, thiazide and tolvaptan.

LVEF, left ventricular ejection fraction; BNP, brain natriuretic peptide; eGFR, estimated glomerular filtration rate; ACE-I, angiotensin-converting enzyme inhibitor; ARB, angiotensin-receptor blocker; MRA, mineralocorticoid receptor antagonist; HR, hazard ratio; CI, confidence interval.
