# Supplementary material for: Changes in BNP levels from discharge to 6-month visit predict subsequent outcomes in patients with acute heart failure
Source: PLoS One. 2022 Jan 28;17(1):e0263165. doi: 10.1371/journal.pone.0263165 (PMC8797237; doi:10.1371/journal.pone.0263165)
Supplement: S3 Table — Values are n/n (%). BNP, brain natriuretic peptide; LVEF, left ventricular ejection fraction; ACE-I, angiotensin-converting enzyme inhibitor; ARB, angiotensin-receptor blocker; HR, hazard ratio; CI, confidence interval. (PDF) [file pone.0263165.s003.pdf]

S3 Table. Subgroup analysis for the primary outcome measure according to the tertiles of percent change in BNP

Subgroups	Categorized group	N of patients with event/N of patients at risk (Cumulative 180-day incidence)	Crude HR (95% CI)	P value	Adjusted HR (95% CI)	P value	P interaction
**BNP at 6-month visit**							
BNP >295 ng/L	BNP worsening	26/86 (30.3 %)	1.53 (0.46-9.44)	0.54	2.68 (0.51-49.4)	0.29	0.82
	No-marked BNP change	9/54 (17.1 %)	0.93 (0.25-5.96)	0.92	1.36 (0.23-25.9)	0.77	
	Marked BNP improvement	2/8 (25 %)	1 (Reference)		1 (Reference)		
295 ng/L ≥BNP >120 ng/L	BNP worsening	10/42 (25.1 %)	1.84 (0.68-5.40)	0.23	1.16 (0.33-4.15)	0.82	
	No-marked BNP change	9/63 (14.3 %)	1.11 (0.41-3.25)	0.84	0.97 (0.28-3.49)	0.96	
	Marked BNP improvement	6/43 (14.6 %)	1 (Reference)		1 (Reference)		
120 ng/L ≥BNP	BNP worsening	4/20 (20 %)	7.00 (1.54-35.6)	0.01	6.16 (0.85-53.1)	0.07	
	No-marked BNP change	3/32 (10.0 %)	3.29 (0.61-17.8)	0.16	2.26 (0.26-21.7)	0.45	

	Marked BNP improvement	2/98 (2.1 %)	1 (Reference)		1 (Reference)		
**Atrial arrythmias**							
Yes	BNP worsening	25/98 (25.6 %)	4.51 (1.74-11.7)	0.002	3.23 (1.07-10.1)	0.04	
	No-marked BNP change	12/102 (12.1 %)	2.11 (0.77-5.80)	0.15	1.49 (0.48-4.62)	0.49	
	Marked BNP improvement	4/69 (5.9 %)	1 (Reference)		1 (Reference)		
No	BNP worsening	14/50 (29.2 %)	4.72 (1.83-12.2)	0.001	2.91 (0.79-10.7)	0.11	0.87
	No-marked BNP change	9/47 (19.4 %)	3.03 (1.10-8.34)	0.03	1.77 (0.39-8.18)	0.46	
	Marked BNP improvement	6/80 (7.6 %)	1 (Reference)		1 (Reference)		
**LVEF <40 %**							
Yes	BNP worsening	13/39 (34.4 %)	6.26 (1.75-39.9)	0.003	7.91 (1.68-60.1)	0.007	
	No-marked BNP change	11/41 (27.0 %)	4.70 (1.28-30.2)	0.02	5.51 (1.21-39.2)	0.03	
	Marked BNP improvement	2/29 (7.0 %)	1 (Reference)		1 (Reference)		0.66
No	BNP worsening	22/100 (22.3 %)	3.79 (1.79-9.00)	0.0003	2.53 (0.96-7.40)	0.06	
	No-marked BNP change	10/96 (10.7 %)	1.59 (0.64-4.10)	0.32	1.07 (0.34-3.37)	0.90	
	Marked BNP	7/108 (6.7 %)	1 (Reference)		1 (Reference)		

	improvement						
**ACE-I or ARB**							
Yes	BNP worsening	18/73 (24.7 %)	5.25 (2.14-15.7)	0.0001	4.11 (1.47-13.6)	0.006	0.048
	No-marked BNP change	13/73 (18.2 %)	3.27 (1.25-10.1)	0.01	1.69 (0.54-5.90)	0.37	
	Marked BNP improvement	4/80 (5.2 %)	1 (Reference)		1 (Reference)		
No	BNP worsening	13/44 (31.2 %)	2.77 (1.04-8.63)	0.04	2.51 (0.67-11.5)	0.18	
	No-marked BNP change	7/47 (15.4 %)	1.40 (0.47-4.64)	0.55	1.45 (0.38-6.25)	0.59	
	Marked BNP improvement	5/39 (12.8 %)	1 (Reference)		1 (Reference)		
**β-blocker**							
Yes	BNP worsening	20/84 (24.4 %)	3.84 (1.78-9.19)	0.0005	3.55 (1.35-10.2)	0.009	0.48
	No-marked BNP change	13/93 (14.3 %)	2.04 (0.87-5.10)	0.10	1.85 (0.69-5.34)	0.22	
	Marked BNP improvement	7/102 (7.1 %)	1 (Reference)		1 (Reference)		
No	BNP worsening	11/33 (33.3 %)	3.83 (1.04-24.6)	0.04	2.44 (0.40-21.1)	0.34	
	No-marked BNP change	6/27 (22.4 %)	2.43 (0.59-16.3)	0.23	1.63 (0.26-13.6)	0.60	
	Marked BNP improvement	2/18 (11.1 %)	1 (Reference)		1 (Reference)		

Values are n/n (%).

BNP, brain natriuretic peptide; LVEF, left ventricular ejection fraction; ACE-I, angiotensin-converting enzyme inhibitor; ARB, angiotensin-receptor blocker; HR, hazard ratio; CI, confidence interval.
