# Supplementary material for: Changes in BNP levels from discharge to 6-month visit predict subsequent outcomes in patients with acute heart failure
Source: PLoS One. 2022 Jan 28;17(1):e0263165. doi: 10.1371/journal.pone.0263165 (PMC8797237; doi:10.1371/journal.pone.0263165)
Supplement: S4 Table — Categorical variables are presented as number (%), and continuous variables are presented as mean ± SD or median (interquartile range). BMI, body mass index; AF, atrial fibrillation; AFL, atrial flutter; ICH, intracranial hemorrhage; BP, blood pressure; HR, heart rate; BNP, brain natriuretic peptide; eGFR, estimated glomerular filtration rate; SD, standard deviation. (PDF) [file pone.0263165.s004.pdf]

S4 Table. Patient characteristics compared between patients with available data on medications at 6-month visit and those without data

Variables	Data available on medication (N=352)	Data unavailable on medication (N=94)	P value
Age (years)	75.0 ± 12.3	74.4 ± 12.6	0.76
Age ≥80 years	153 (43 %)	42 (45 %)	0.83
Women	160 (45 %)	49 (52 %)	0.25
BMI (kg/m^2)	22.8 ± 4.9	23.4 ± 5.0	0.32
BMI ≤22 kg/m^2	133 (47 %)	33 (46 %)	0.82
AF or AFL	218 (62 %)	51 (54 %)	0.18
Hypertension	258 (73 %)	69 (73 %)	0.98
Diabetes	129 (37 %)	37 (39 %)	0.63
Previous myocardial infarction	76 (22 %)	25 (27 %)	0.31
BNP at discharge (ng/L)	244 (114-450)	211 (139-391)	0.47
BNP at 6-month visit (ng/L)	184 (82.4-396)	158 (76.6-358)	0.62
Change in BNP (ng/L)	-19.9 (-166-73.0)	-7.9 (-119-86.4)	0.55
% change in BNP (%)	-15.1 (-61.2-44.4)	-7.5 (-57.0-51.6)	0.83
eGFR (mL/min/1.73m^2)	45.2 ± 20.7	45.5 ± 18.4	0.57
eGFR <30 mL/min/1.73m^2	87 (25 %)	21 (22 %)	0.62
Albumin (g/dL)	3.9 ± 0.52	4.0 ± 0.61	0.005
Albumin <3 g/dL	14 (4.2 %)	3 (3.3 %)	0.68
Hemoglobin (g/dL)	11.9 ± 2.0	12.2 ± 2.4	0.13

Categorical variables are presented as number (%), and continuous variables are presented as mean ± SD or median (interquartile range).

BMI, body mass index; AF, atrial fibrillation; AFL, atrial flutter; ICH, intracranial hemorrhage; BP, blood pressure; HR, heart rate; BNP, brain natriuretic peptide; eGFR, estimated glomerular filtration rate; SD, standard deviation.
