# Supplementary figures and images for: Changes in BNP levels from discharge to 6-month visit predict subsequent outcomes in patients with acute heart failure
Source: PLoS One. 2022 Jan 28;17(1):e0263165. doi: 10.1371/journal.pone.0263165 (PMC8797237; doi:10.1371/journal.pone.0263165)

S1 Fig. Histogram of percent change in BNP from discharge to 6-month visit.

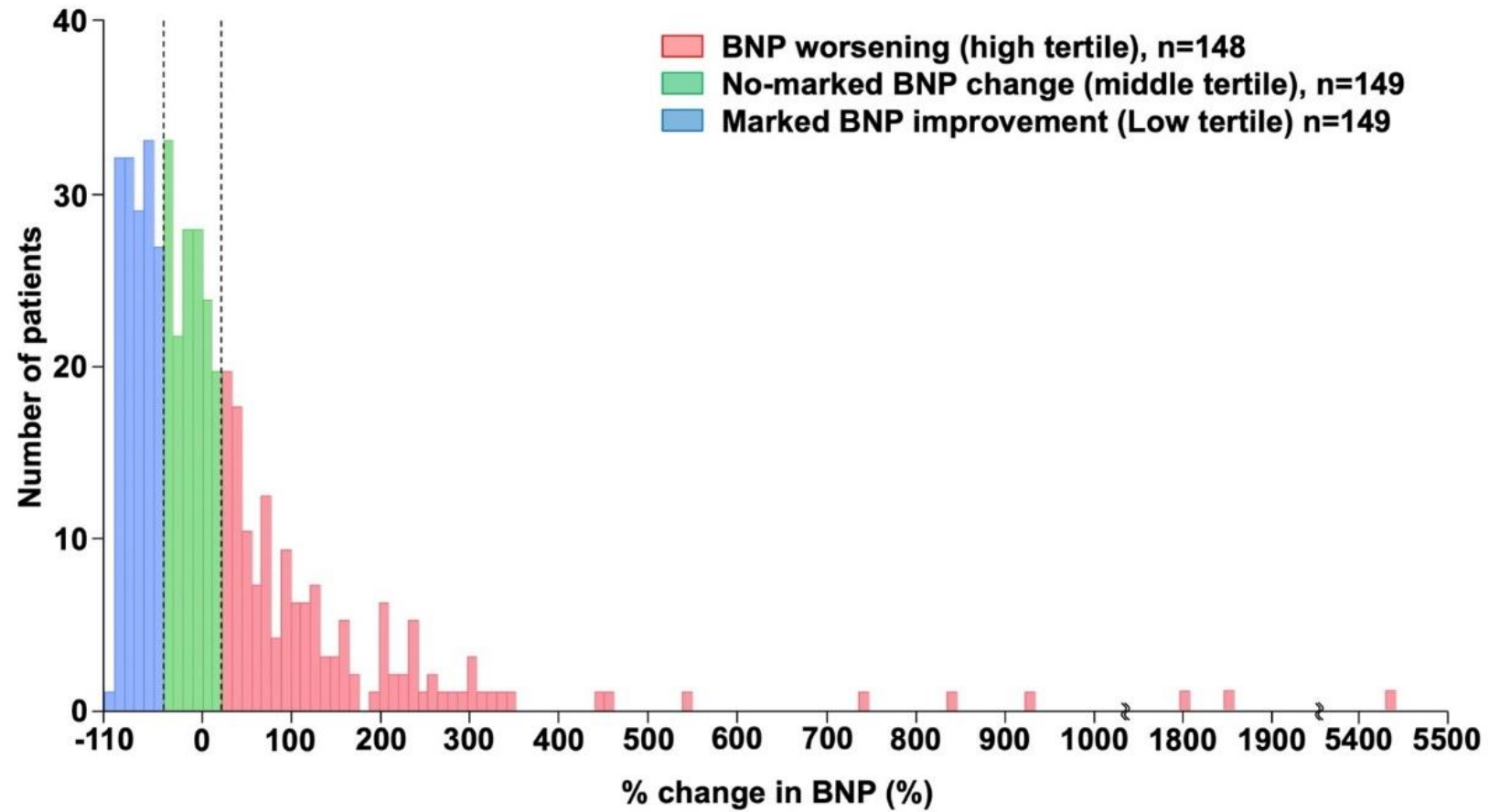

Supplement: S1 Fig — % change in BNP, percent change in brain natriuretic peptide. (PDF) [file pone.0263165.s005.pdf]
