# Supplementary material for: Changes in BNP levels from discharge to 6-month visit predict subsequent outcomes in patients with acute heart failure
Source: PLoS One. 2022 Jan 28;17(1):e0263165. doi: 10.1371/journal.pone.0263165 (PMC8797237; doi:10.1371/journal.pone.0263165)
Supplement: S2 Fig — This study population was classified into the 3 groups by percent change in BNP during discharge and 6-month visit; the marked BNP improvement group (≤-44%, N = 149), the no-marked BNP change group (>-44% and ≤22%, N = 149) and the BNP worsening group (>22%, N = 148). Red lines indicate patients with events. Blue lines indicate patients without events. BNP, brain natriuretic peptide; HF, heart failure. (PDF) [file pone.0263165.s006.pdf]

S2 Fig. Changes in BNP during discharge and 6-month visit in (A) the marked BNP improvement group, (B) the no-marked BNP change group, and (C) the BNP worsening group.

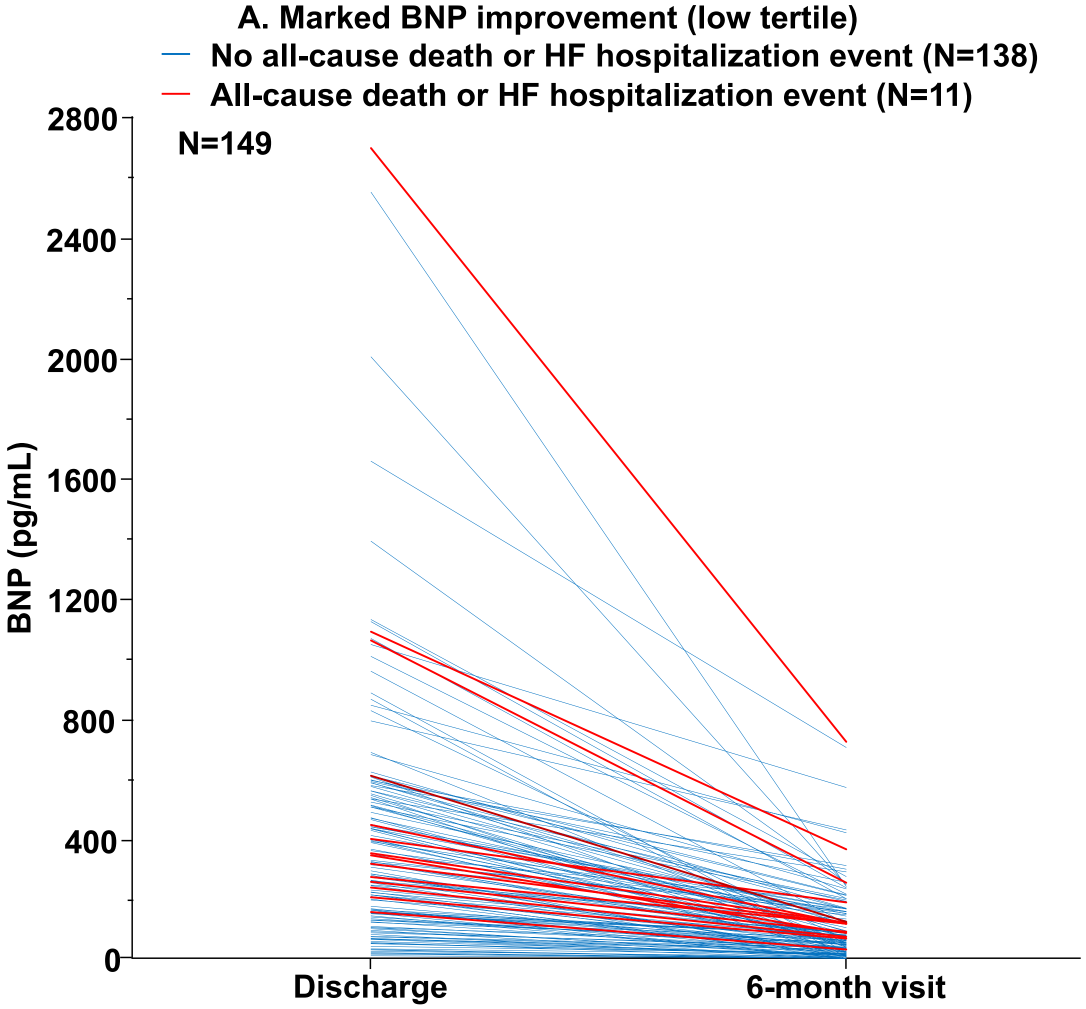

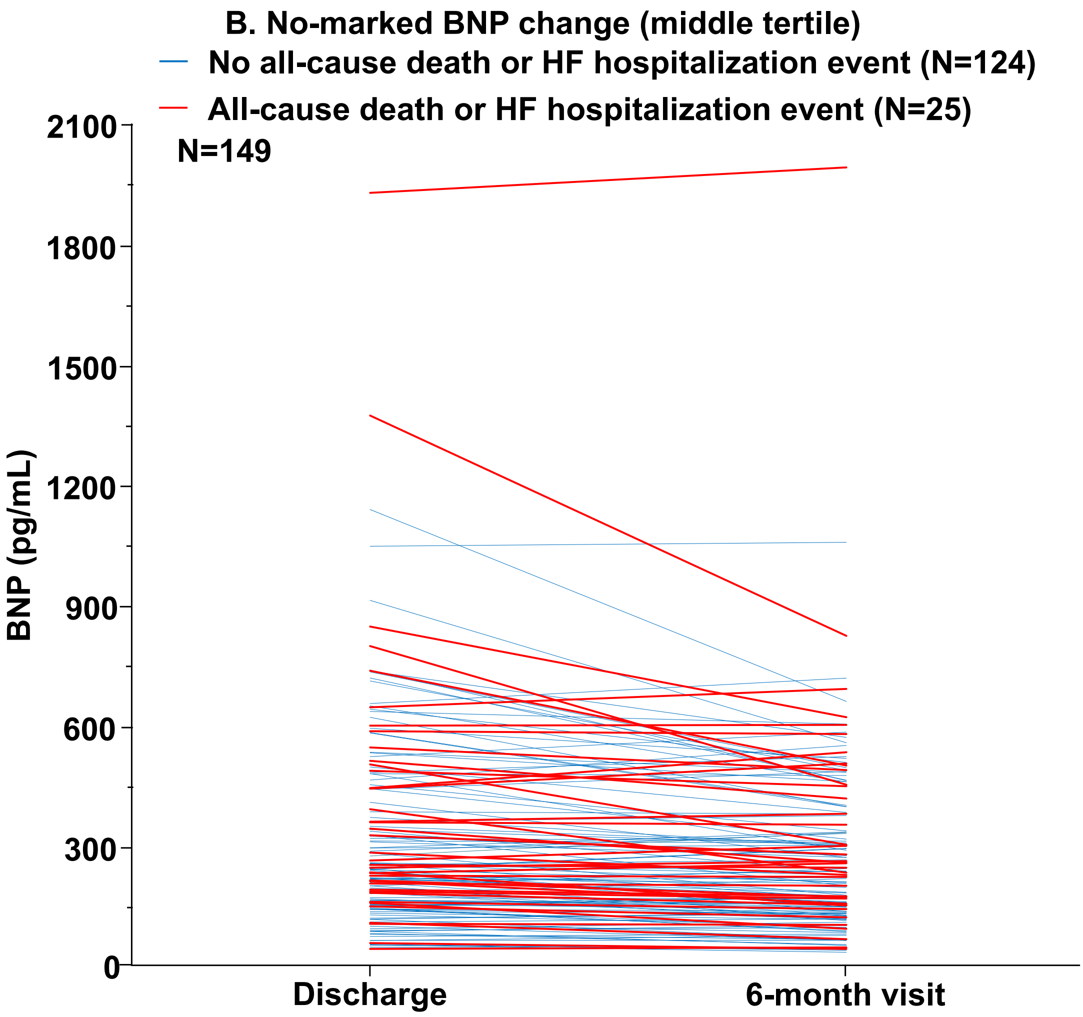
B. No-marked BNP change (middle tertile)
No all-cause death or HF hospitalization event (N=124)
All-cause death or HF hospitalization event (N=25)
N=149
2100
1800
1500
1200
900
600
300
0
BNP (pg/mL)
Discharge
6-month visit

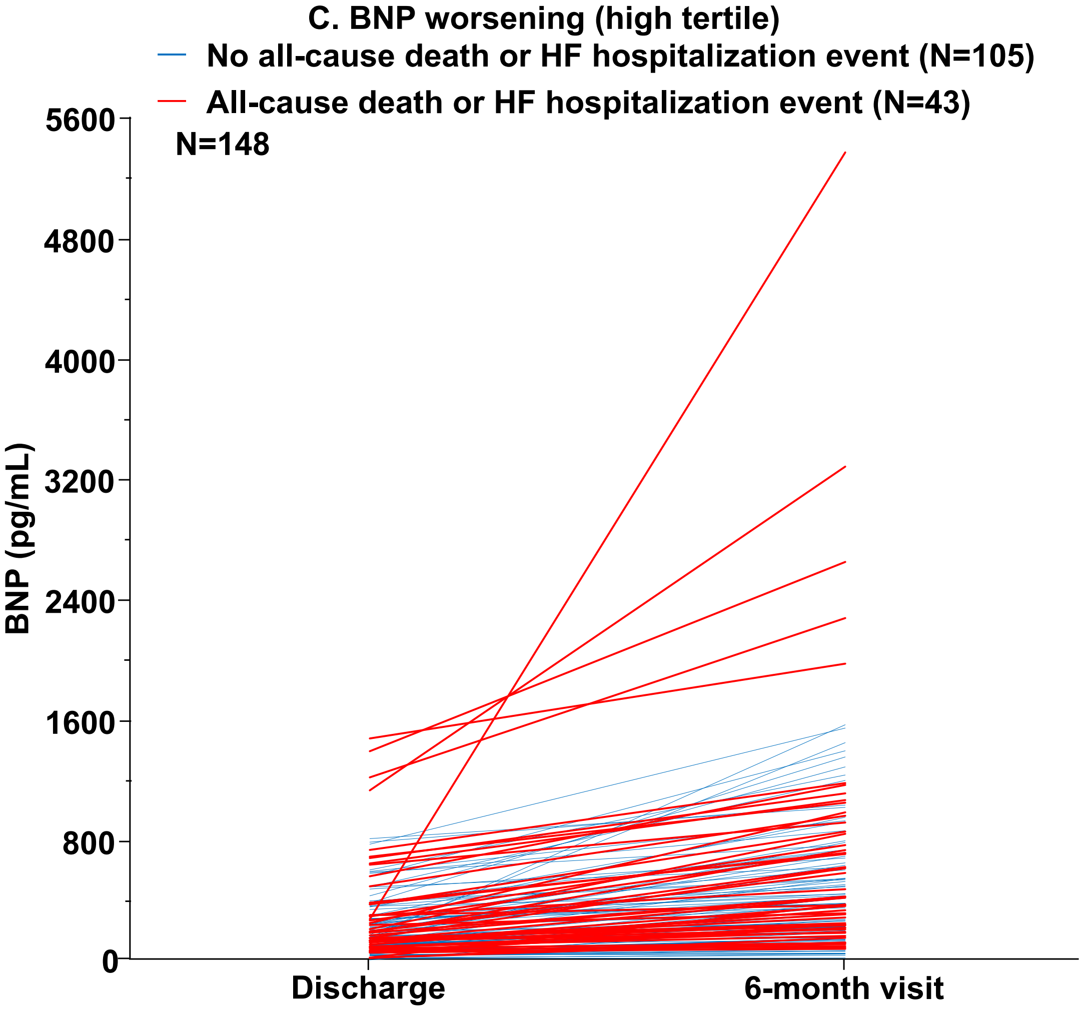

This study population was classified into the 3 groups by percent change in BNP during discharge and 6-month visit; the marked BNP improvement group (≤-44 %, N=149), the no-marked BNP change group (>-44 % and ≤22 %, N=149) and the BNP worsening group (>22 %, N=148). Red lines indicate patients with events. Blue lines indicate patients without events.

BNP, brain natriuretic peptide; HF, heart failure.
