# Supplementary material for: Changes in BNP levels from discharge to 6-month visit predict subsequent outcomes in patients with acute heart failure
Source: PLoS One. 2022 Jan 28;17(1):e0263165. doi: 10.1371/journal.pone.0263165 (PMC8797237; doi:10.1371/journal.pone.0263165)
Supplement: S3 Fig — The primary outcome measure was defined as a composite of all-cause death or hospitalization for heart failure. BNP, brain natriuretic peptide. (PDF) [file pone.0263165.s007.pdf]

S3 Fig. Kaplan Meier curves for the primary outcome measure in three subpopulations according to BNP level at discharge.

A. Low tertile of BNP at discharge (≤155 pg/mL, N=150)

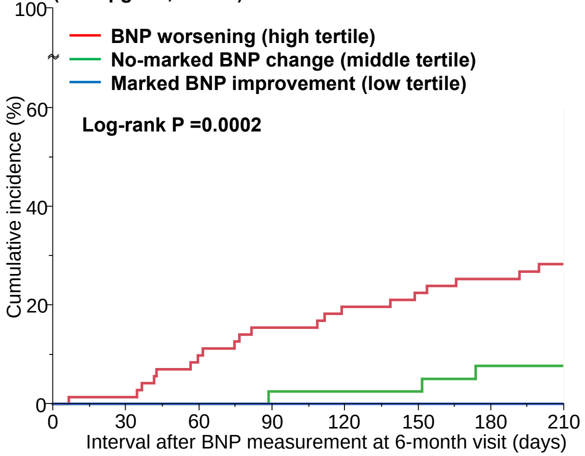

Interval (days)	0	60	120	180
**BNP worsening (high tertile)**				
N of patients with at least 1 event		7	14	18
N of patients at risk	73	65	58	52
Cumulative incidence		9.8 %	19.7 %	25.3 %
**No-marked BNP change (middle tertile)**				
N of patients with at least 1 event		1	1	3
N of patients at risk	41	40	39	35
Cumulative incidence		2.5 %	2.5 %	7.7 %
**Marked BNP improvement (low tertile)**				
N of patients with at least 1 event		0	0	0
N of patients at risk	36	36	36	35
Cumulative incidence		0 %	0 %	0 %

B. Middle tertile of BNP at discharge (>155 pg/mL and ≤350 pg/mL, N=147)

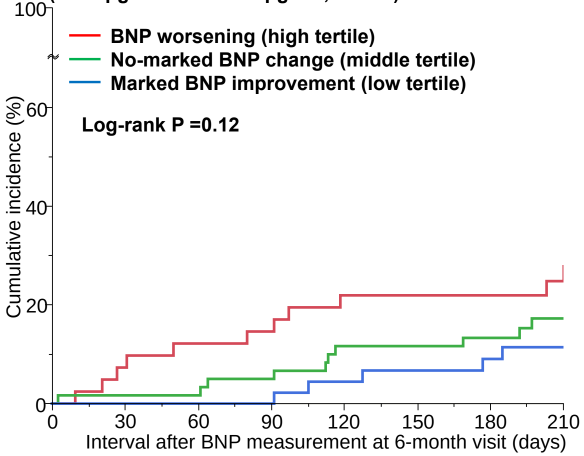

Interval (days)	0	60	120	180
**BNP worsening (high tertile)**				
N of patients with at least 1 event		5	9	9
N of patients at risk	41	37	33	30
Cumulative incidence		12.2 %	22.0 %	22.0 %
**No-marked BNP change (middle tertile)**				
N of patients with at least 1 event		2	7	8
N of patients at risk	60	59	54	48
Cumulative incidence		3.3 %	11.7 %	13.3 %
**Marked BNP improvement (low tertile)**				
N of patients with at least 1 event		1	3	4
N of patients at risk	46	45	42	39
Cumulative incidence		2.2 %	6.7 %	9.1 %

C. High tertile of BNP at discharge (>350 pg/mL, N=149)

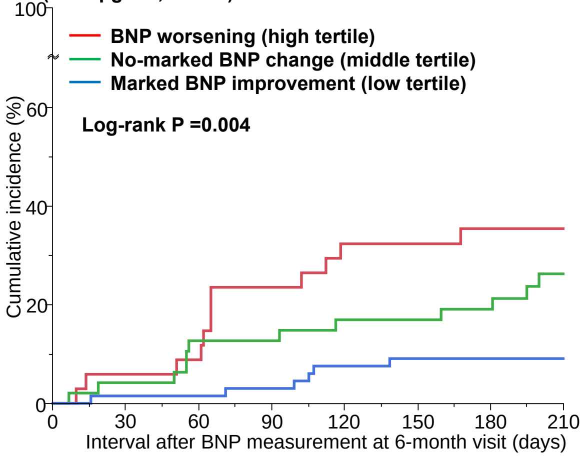

Interval (days)	0	60	120	180
**BNP worsening (high tertile)**				
N of patients with at least 1 event		4	11	12
N of patients at risk	34	31	24	21
Cumulative incidence		11.8 %	32.4 %	35.4 %
**No-marked BNP change (middle tertile)**				
N of patients with at least 1 event		6	8	10
N of patients at risk	48	42	40	37
Cumulative incidence		12.7 %	16.9 %	21.3 %
**Marked BNP improvement (low tertile)**				
N of patients with at least 1 event		1	5	6
N of patients at risk	67	66	62	55
Cumulative incidence		1.5 %	7.6 %	9.1 %

The primary outcome measure was defined as a composite of all-cause death or hospitalization for heart failure.

BNP, brain natriuretic peptide.
